# Towards an understanding of the chemo-mechanical influences on kidney stone failure via the material point method

**DOI:** 10.1371/journal.pone.0240133

**Published:** 2020-12-11

**Authors:** Samuel J. Raymond, Janille Maragh, Admir Masic, John R. Williams

**Affiliations:** 1 Department of Civil and Environmental Engineering, Massachusetts Institute of Technology, Cambridge, MA, United States of America; 2 Center for Computational Science and Engineering, Massachusetts Institute of Technology, Cambridge, MA, United States of America; University of Zaragoza, SPAIN

## Abstract

This paper explores the use of the meshfree computational mechanics method, the Material Point Method (MPM), to model the composition and damage of typical renal calculi, or kidney stones. Kidney stones are difficult entities to model due to their complex structure and failure behavior. Better understanding of how these stones behave when they are broken apart is a vital piece of knowledge to medical professionals whose aim is to remove these stone by breaking them within a patient’s body. While the properties of individual stones are varied, the common elements and proportions are used to generate synthetic stones that are then placed in a digital experiment to observe their failure patterns. First a more traditional engineering model of a Brazil test is used to create a tensile fracture within the center of these stones to observe the effect of stone consistency on failure behavior. Next a novel application of MPM is applied which relies on an ultrasonic wave being carried by surrounding fluid to model the ultrasonic treatment of stones commonly used by medical practitioners. This numerical modeling of Extracorporeal Shock Wave Lithotripsy (ESWL) reveals how these different stones failure in a more real-world situation and could be used to guide further research in this field for safer and more effective treatments.

## 1 Introduction

Nephrolithiasis, the growth of kidney stones inside the human body, is one of the most common urological conditions. In the US alone, [1] estimates that over 10% of the male population and over 7% of the female population suffer from kidney stones. There is also evidence that this trend is likely to increase in severity in many parts of the developed world [1, 2]. Treatment of these stones has been the focus of several decades of modern research [3–5] with a focus on the now commonly used treatment of Extracorporeal Shock Wave Lithotripsy (ESWL). This method uses ultrasonic waves to pulse the stones inside the kidney with high frequency pressure waves, inducing various failure mechanisms within the material [5, 6]. Additional directions of research such as the formation of renal stones [7] and the properties of the micro-scale structures that are thought to seed kidney stones [8], highlight the lack of full understanding of these near ubiquitous stones. Kidney stones’, and gallstones’, mechanical properties have been difficult to measure but some research [4, 9, 10] has attempted to measure these properties and create artificial stones [10] so that they can be tested in experiments. In these cases the material was seen as a single phase with the properties borne out of the complex chemical structure that is the result of the stone formation.

To model the behavior of these stones, several frameworks have been applied to understand the fragmentation of these stones under conditions like ESWL. In [11], a theoretical framework for kidney stones with a random micro structure was presented. Stress concentrations were added to the stress description to describe the effect of shock waves on a kidney stone. [6] applied different shock wave experiments on plaster of Paris stones to observe the role of stress waves and cavitation in shock wave lithotripsy in kidney stones. In this work the stress waves and cavitation were found to work together rather than independently to produce the fragmentation of the stone. An investigation into the mechanisms of stone failure via spall damage was conducted by [12]. This theoretical model used cohesive zones to analyze the role of this mechanism in stone failure. However, these theoretical approaches fall short of direct information for engineering solutions to this problem.

Computational modeling of the damage and fragmentation of kidney stones is a powerful tool in helping to understand the behavior of these stones and to aid in better treatment. Many different studies have been attempted in the past to simulate the failure of these stones. In [13] the PDS-FEM method was used to study 3D dynamic crack propagation in conjunction with experimental observation of Shock Wave Lithotripsy (SWL). They showed that dominate stone fragmentation is controlled by the high amplitude shear waves. Crack patterns were compared between the simulation and the experimental results. While these results showed good agreement between the model and the experiment, the kidney stone was represented as a simple cylinder of plaster of Paris. In an attempt to understand the role of the shock wave, [14] used a time-dependent Finite Difference solution to model the waves present in the SWL of kidney stones. Shear wave interference from the outer edge of the stone was shown to lead to the peak loading. However, no damage model was included and this also relied on a simple representation of a stone material. An analysis of the mechanics of shock wave on a stone was studied using Finite Difference by [15]. In this work, hypotheses were tested to determine different mechanisms that could be possible for failure as the sole mechanism. However, no single mechanism was found to be the direct cause of failure. Fracture modeling was not included in the numerical model but it was concluded that all of the failure mechanisms contributed to the overall fragmentation. In [16] a damage model was proposed for kidney stones based on the Tuler-Butcher concept. A Finite Volume Method (FVM) simulation was used to simulate this model with the proposed damage model and compared with statistical crack distributions obtained from experiments using BegoStone samples. Good agreement between the damage patterns predicted and those from experiments were shown. However fragmentation was not permitted and the material was also modeled as a single material. [17] used a gypsum cylinder with a Finite Element Method (FEM) model and loaded to fracture to emulate a kidney stone. Another FEM simulation of kidney stones under impact was used to study the fragmentation behavior in [18]. A cohesive zone model for damage was used to allow separation of the FEM elements. In their work they varied the indenter shape and investigated the effect of multiple impacts the fragmentation. An issue with mesh-based techniques, however, is the complications that arise in areas of damage/fracture growth where the elements require special treatment.

In this work, instead of treating the material of the kidney stone as a single phase, the chemical composition and distribution on a macro-scale of these stones is included in a numerical model so that the mechanical properties can be influenced by the geometrical distributions of common stone chemistry. To simulate the fracture of a stone with this complex structure, the meshfree particle method, the Material Point Method (MPM), which has been used to study the effects of other rock and aggregate materials [19] is applied to kidney stones. The damage model allows for fragmentation of these stones to be handled simply in the method without complex multi-step approaches and combined numerical methods. These stones’ properties are informed from the experimental analysis of the chemical composition of real stones. These properties and their distribution are used to create synthetic stones to be used in the simulation. This method allows us to study the effect of real stone compositions and the resultant fracture behavior. Numerical models experiments used to test a brittle materials fracture strength are performed on these synthetic digital stone geometries to observe the effect of stone morphology on failure behavior.

## 2 Methods

In this section the tools used to create the models are presented. Firstly the chemical characterization technique, energy dispersive x-ray spectroscopy (EDS), is presented. Secondly, the mechanical testing technique, nanoindentation, is presented. Thirdly, the numerical technique, MPM is introduced breifly, with a description of the technique and uses in other fields of science and engineering.

### 2.1 Chemo-mechanical characterization of kidney stone structure

#### 2.1.1 Chemical mapping of kidney stones

Many characterization techniques introduce inconvenient challenges, such as the need for coated polished sections in standard scanning electron microscopy (SEM), the inability to spatially resolve phases as in bench-top X-ray diffraction, and the low contrast between different phases typical in the study of cements using SEM/BSE imaging data [20, 21]. In previous characterization studies of kidney stones, infrared (IR) spectroscopy has been used to perform chemical mapping, but the resulting maps were either poor in resolution [22–24] due to the long wavelengths used for imaging, or involved the use of synchrotron radiation [25]. As such, low vacuum EDS, in which samples need not be coated with a conductive layer, was used to quantitatively map the elements present in a polished cross-section of a kidney stone consisting of calcium oxalate monohydrate, uric acid, and proteins. Low-vacuum environmental SEM-EDS mapping requires minimal sample preparation and is able to spatially resolve phases and elements with high compositional specificity, limited only by the physical limit of the interaction volume of the electron beam with the sample. Furthermore, with the development of multi-detector EDS, in which two diametrically opposed detected are used to detect x-rays from the sample’s surface, it is also possible to obtain quantitative elemental information for three-dimensional surfaces. This has made it an apt choice for the study of complex composites, including Roman concrete and sea urchin teeth, particularly when combined with other complementary characterization techniques such as Raman spectroscopy [26, 27]. The high throughput nature of EDS mapping has made it possible to combine quantitative EDS data with clustering techniques to map the distribution of chemically distinct phases throughout the mapped area of a sample [28].

#### 2.1.2 Microscale-mechanical properties of kidney stones

The Oliver-Pharr indentation method was developed to measure the elastic modulus (M) and hardness (H) of localized regions of materials [29], and over the years, the technique continues to be further refined and enhanced [30]. The technique was later extended to allow for the measurement of the local creep modulus (C) through the study of the logarithmic creep compliance curve obtained from a minutes-long indent [31]. Nanoindentation has been used in the characterization of a wide variety of materials. It has been used to study optical micro-electro-mechanical systems to observe the variations in elastic modulus and hardness among different layers [32]. It has also been used at length in recent years for the characterization of micro structural elements and very small material features, such as thin films [33–37]. Whereas nanoindentation is often conducted on distinct areas, such as in the micromechanical characterization of bone phases that are distinguishable in SEM images [38], nanoindentation grid mapping allows for the collection of statistical mechanical information. A previous study has shown that by deconvoluting the multi modal nanoindentation grid mapping data of multi-phase materials, the volume fractions and average mechanical properties of the individual phases can be obtained [39]. Therefore, when nanoindentation grid mapping is performed on a single phase, the H, M, and C data extracted from the indentation curves is normally distributed, and the means of the distributions represent the average H, M, and C values for that phase. In a three-dimensional composite, mechanical properties obtained using indentation is likely not attributed to the single phase observed on the two-dimensional cross-section. Rather, since phases lie on top of one another, there will likely be a contribution from another phase beneath it. Because of imperfections such as these, the mechanical properties obtained via the grid mapping of a “single phase” will be observed to be normally distributed [39].

#### 2.1.3 Mapping properties of kidney stones for modeling

**Fig 1:**
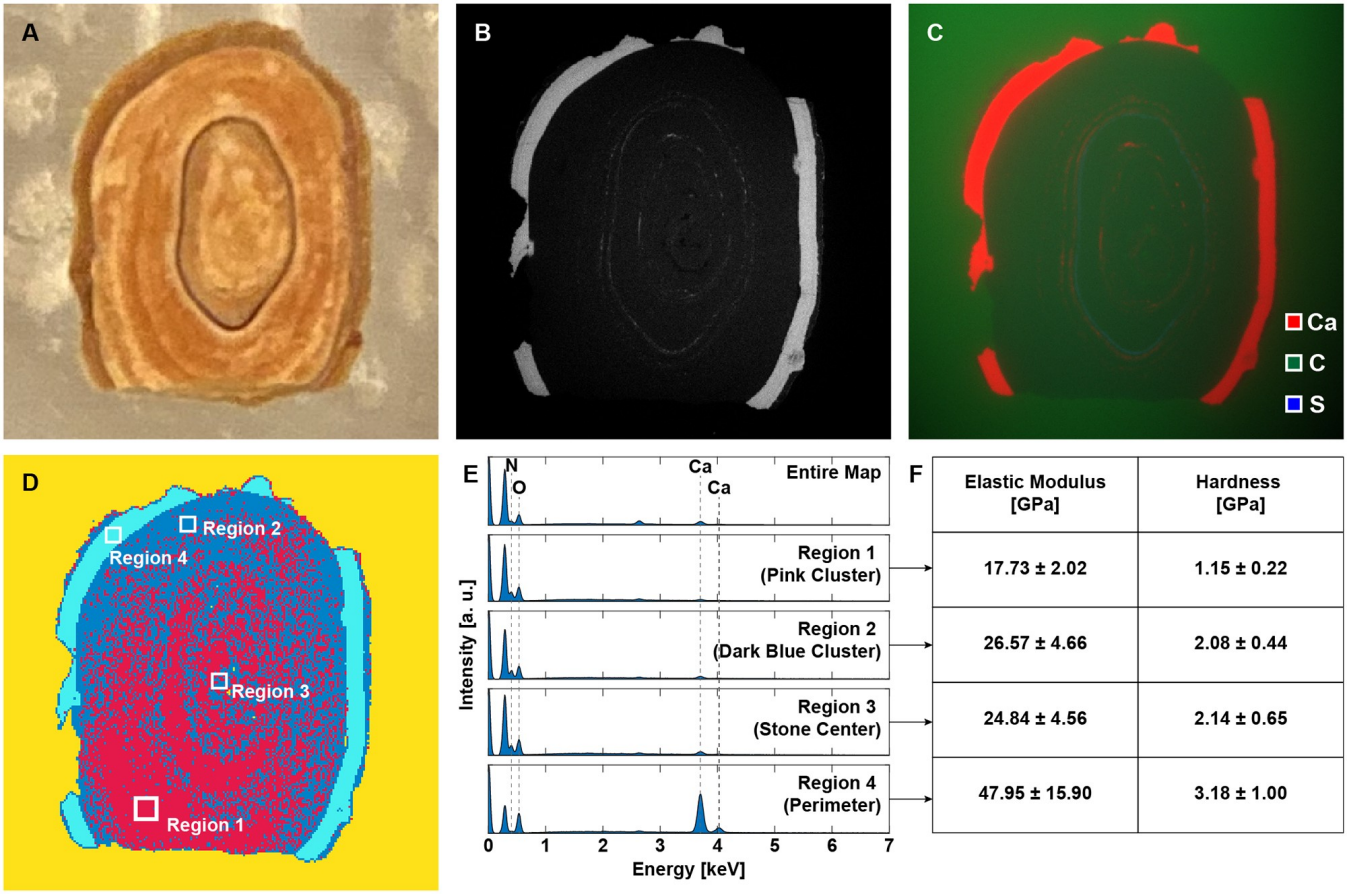
Chemomechanical characterization of the polished cross-section of a calcium oxalate monohydrate, uric acid, and protein kidney stone. (A) Photograph of cross-section. (B) BS-SEM image. (C) SEM-EDS map showing distribution of calcium, carbon, and sulfur. (D) Spatial distribution of EDS clusters obtained using fuzzy c-means clustering. (E) EDS spectra for the entire map and each of the four regions indicated in (D). (F) Mean values and standard deviations for the elastic moduli and hardness values for each of the four regions indicated in (D) obtained using nanoindentation grid mapping.

SEM-EDS element mapping was conducted using a Tescan Vega scanning electron microscope equipped with two diametrically opposed energy dispersive spectrometers with individual detector areas of 30 mm2 (Bruker XFlash 5030). The back-scattered electron (BSE) micrograph was acquired with a YAG crystal scintillator-based detector at 16-bit image resolution, resulting in 65,536 distinct grayscale intensity values. The quantitative element map was then segmented using fuzzy c-means clustering to obtain the spatial distribution of chemically distinct phases. Nanoindentation grid mapping was then performed on each phase distinguishable in the clustered map using the CSM Instruments standard nanoindentation tester. Analysis of the data from each indent yielded the elastic modulus, hardness, and creep modulus at that point of the sample’s surface, and for each of the four regions of the kidney stone tested using nanoindentation grid mapping (Fig 1), 625 nanoindentations were conducted in 25-by-25 grids. Fig 1 shows the chemomechanical analysis of a kidney stone consisting of calcium oxalate monohydrate, uric acid, and proteins. The layered structure of the kidney stone is identifiable in the photograph of the cross section in Fig 1A. The photograph of the kidney stone used for the chemomechanical analysis shows its layered structure (Fig 1A). SEM-EDS element mapping (Fig 1C) was conducted using a Tescan Vega scanning electron microscope equipped with two diametrically opposed energy dispersive spectrometers with individual detector areas of 30 mm2 (Bruker XFlash 5030). The back-scattered electron (BSE) micrograph (Fig 1B) was acquired with a YAG crystal scintillator-based detector at 16-bit image resolution, resulting in 65,536 distinct grayscale intensity values. The quantitative element map was then segmented using fuzzy c-means clustering to obtain the spatial distribution of chemically distinct phases (Fig 1D). Nanoindentation grid mapping was then performed on each phase distinguishable in the clustered map using the CSM Instruments standard nanoindentation tester. Analysis of the data from each indent yielded the elastic modulus, hardness, and creep modulus at that point of the sample’s surface, and for each of the four regions of the kidney stone (indicated in Fig 1D) tested using nanoindentation grid mapping (Fig 1), 625 nanoindentations were conducted in 25-by-25 grids. The EDS spectra for the regions indicated in Fig 1D are shown in Fig 1E, and the average H and M values and standard deviations for each of the 4 sub-regions are shown in the table in Fig 1F. Force-depth curves were used to compute the elastic modulus and hardness using the proprietary indentation software Indentation v6.2.9 after manually selecting the contact point for each indent. The creep curves were then used to compute the creep modulus using custom MATLAB software.

### 2.2 The material point method

**Fig 2:**
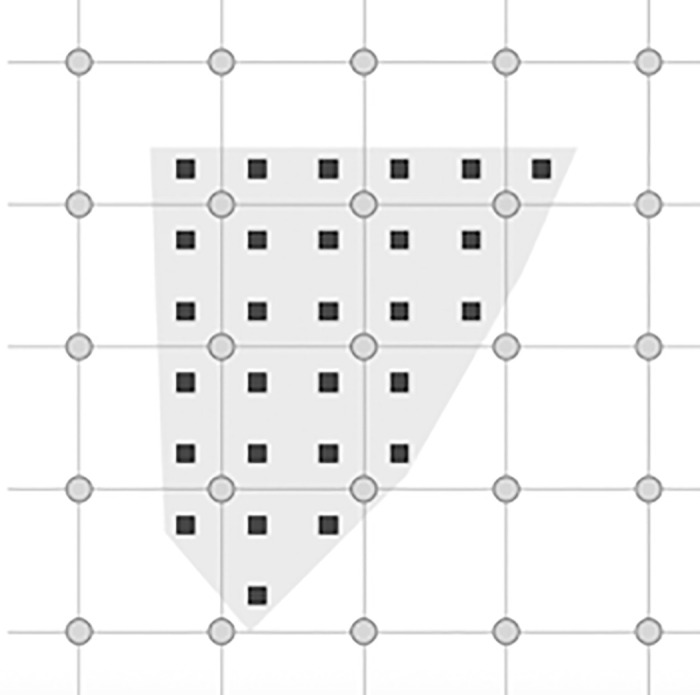
In MPM a material is described by the particles or material points that also hold all of the state variables. Calculation of the derivative terms, such as the current acceleration or strain rate are calculated by using the background grid that the material points can free move around in.

In the past, numerical solutions for governing equations of motions depended on the frame of reference that the equations were derived from. The spatial or Eulerian framework traditionally involves a number of non-linear advection terms in the momentum equation. While this can be used very successfully in certain techniques like the Finite Volume Method and other CFD approaches, these non-linearities can become difficult to work with. Alternatively, the material or Lagrangian framework avoids such analytical features, the difference in this approach is that each point is tracked explicitly from some initial configuration. While this method works best for solids and history-dependent variables (like plasticity), having to track the path of motion often leads to distortion of the mesh in highly deformed materials. Expensive, and often dissipative techniques, like re-meshing in FEM, have been used to deal with mesh distortions but it still represents an inefficient solution. The Material Point Method overcomes the issues associated with both of these frameworks by adopting a solution strategy that utilizes both an Eulerian and Lagrangian perspective. While other methods, such as the Arbitrary Lagrangian-Eulerian (ALE), have been proposed, MPM (and the other PIC methods) is unique in its approach. In MPM the geometry is discretized using material points, to act as a Lagrangian description of a body. These material points are then surrounded by an Eulerian mesh (see Fig 2). The two structures, the material points and the mesh, are entirely independent of each other, they communicate using simple interpolation functions. During the MPM time step, these interpolation, or shape, functions are used to transfer data from the material points to the mesh nodes. Some equations of motion are solved on the mesh nodes and the results are sent back to the material points where the constitutive relationships for each material are solved on the material points. This way, MPM combines both Eulerian and Lagrangian frameworks in order to avoid the common downsides of working with either framework exclusively. This is a key advantage of MPM over other techniques.

#### 2.2.1 Discrete equations of motion of a continua

MPM is, in its fundamental motivation, a method of solving the equations of motion in continuum mechanics. A derivation of this is shown in [19, 40] from the strong form of the equation of motion to the weak form, which is similar to most computational techniques. The unique MPM features begin with the notion of the mass of a single material point. But suffice to say that it is a piece of material, that when combined with a large number of other material points, accurately describes a continuum body. In MPM each material point is defined initially by assigning it a mass, *m*_*p*_, the density is then calculated for the material by:

$$\begin{array}{c} \rho \left(x\right)=\sum_{p}^{N_{mp}}m_{p}\Psi_{p}\left(x\right) \end{array}$$ (1)

In Eq 1, *N*_*mp*_ is the number of material points that constitute the material for which *ρ* is the density, and *Ψ*_*p*_ is a characteristic function of the material point representing the ‘size’ of the material point. In MPM the material point description keeps track of the individual mass and volume of the material point (in most cases) and so this density calculation is not useful. What it is used for is in transforming the integral equations in the weak form, into discrete summations. At this point it is necessary to introduce the background mesh. The nodes of this mesh are assigned interpolation functions that will be used to transmit data to and from the material points. If the nodal interpolation or ‘shape’ functions are denoted as *N*_*i*_ for node ‘i’, then the variable associated with the nodes are described as follows.

$$\begin{array}{c} f\left(x\right)=\sum_{i}^{Nn}f_{i}\left(x\right)N_{i}\left(x\right) \end{array}$$ (2)

In order to translate material point values to nodal values, a unique aspect of MPM arises. As mentioned above the characteristic function, *ψ*_*p*_ of the material point carries information about the shape of the material point, this function must then be multiplied by the nodal shape function, *N*_*i*_, in order to obtain the correctly weighted value, *S*_*ip*_.

$$\begin{array}{c} S_{ip}=N_{i}\left(x_{i},x_{p}\right)\Psi_{p}\left(x_{i},x_{p}\right) \end{array}$$ (3)

This aspect of MPM was not present in the original formulation [41], but has been introduced, generalized [42] and now pervades most of the literature [43] that it is worth introducing at this stage. This follows the trend in many particle-based methods, like SPH [44]. This allows Newton’s 2nd Law for the MPM nodes to be written as follows.

$$\begin{array}{c} \sum_{i}^{N_{n}}m_{ij}{\text{a}}_{i}={\text{f}}_{i}^{int}+{\text{f}}_{i}^{ext} \end{array}$$ (4)

In the above equation, *m*_*ij*_ is the mass matrix for the node, *f*^*int*^ is the internal force vector at the node and *f*^*ext*^ contains the external forces on the node. The forms of these values will be presented next when discussing the MPM time step.

#### 2.2.2 Core MPM time step description

The MPM time-step can be broken into 4 steps. Let there be already in place a discretized domain with material points defining some material undergoing some deformation or stress. Then the following steps will be performed until some predefined condition has been met:

- Extrapolation of material point information to the surrounding grid nodes to form necessary nodal values.
- Solving of equations of motion for the nodes. This is sometimes referred to as the “Lagrangian phase” as it corresponds to solving updated Lagrangian dynamics.
- Updating of material point variables (stress, strain, velocity, position, etc.) using extrapolated nodal values. Sometimes referred to as the “advection phase” since the material points are advecting through the grid (once the next step is completed).
- Resetting (or reformatting) of the surrounding grid.

It is important to note that the function used to represent the material points is generally not the Dirac delta function but instead is a finite-width rectangle. This comes as a result of the work shown in [42] that showed that the delta function representation was not suitable in problems involving stress waves. The Generalized Interpolation Material Point Method, or GIMP, is an extension to this where the Dirac delta function is only a special case. Most commonly a rectangular square with the size of half a cell size is used, this is the approach used in this work. More details into the other functions used and the effect of different sizes of rectangles can be found in the following works [42, 43, 45].

## 3 Results

A key goal of this work is to present a new way of representing the kidney stone in order to observe the failure and fragmentation that occurs when these stones are treated by medical professionals. 2D plane strain assumptions are used to model the stress updates for this and all of the results shown. In addition, while fracture growth is typically a 3D problem, the dominant structures that can be solved in 2D allow for a comparison between different geometries and materials, which is the focus of this work. More complex 3D modeling of fracture initiation and propagation is not covered in the models shown here. The results shown in this section aim to develop progressively more realistic attempts to mimic the real-world behavior of these stones under loading, by implementing more realist material and geometric heterogeneity.

### 3.1 Compression of a kidney stone with the Brazil test

**Fig 3:**
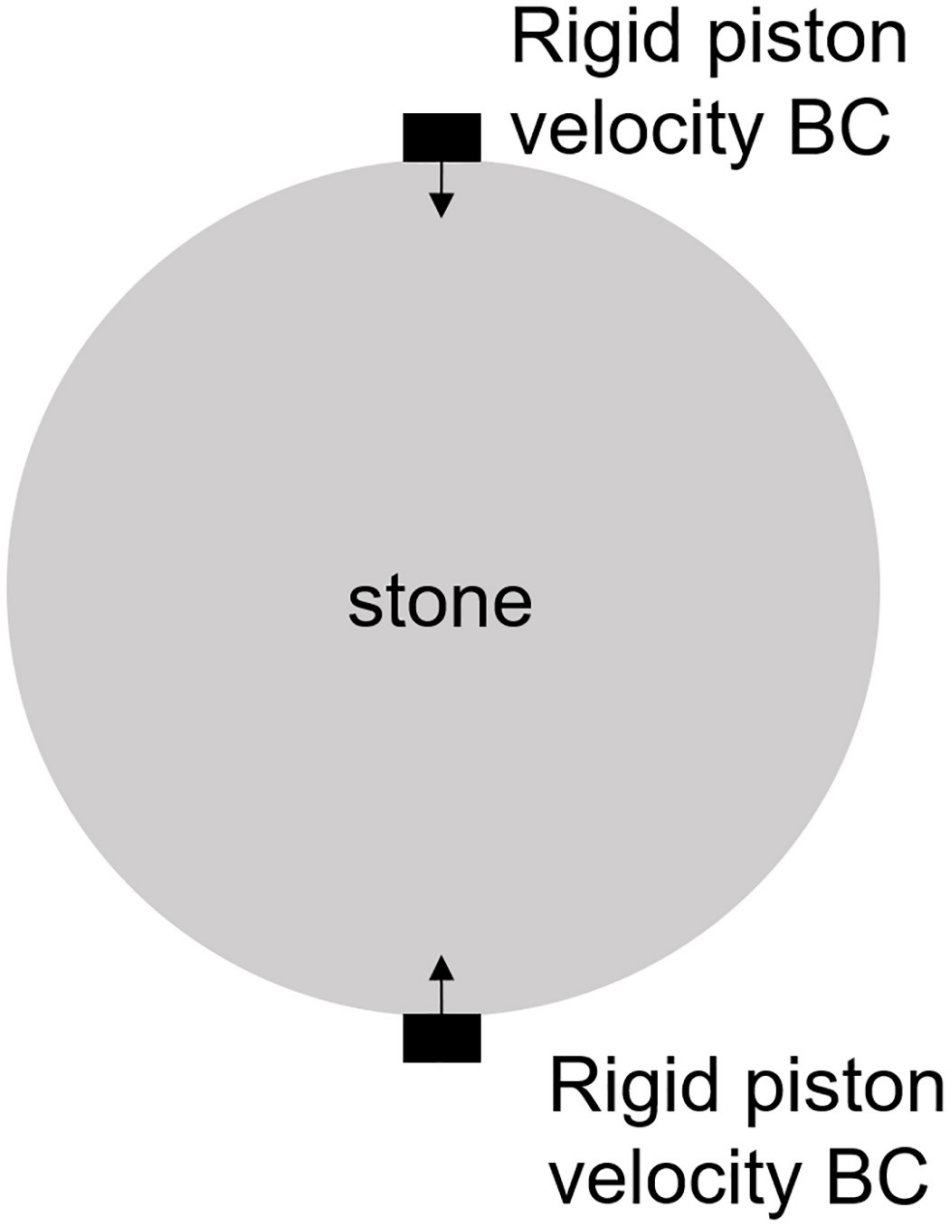
Schematic of the Brazil test where two rigid pistons move with constant velocity to compress the stone to tensile failure along the center line.

**Fig 4:**
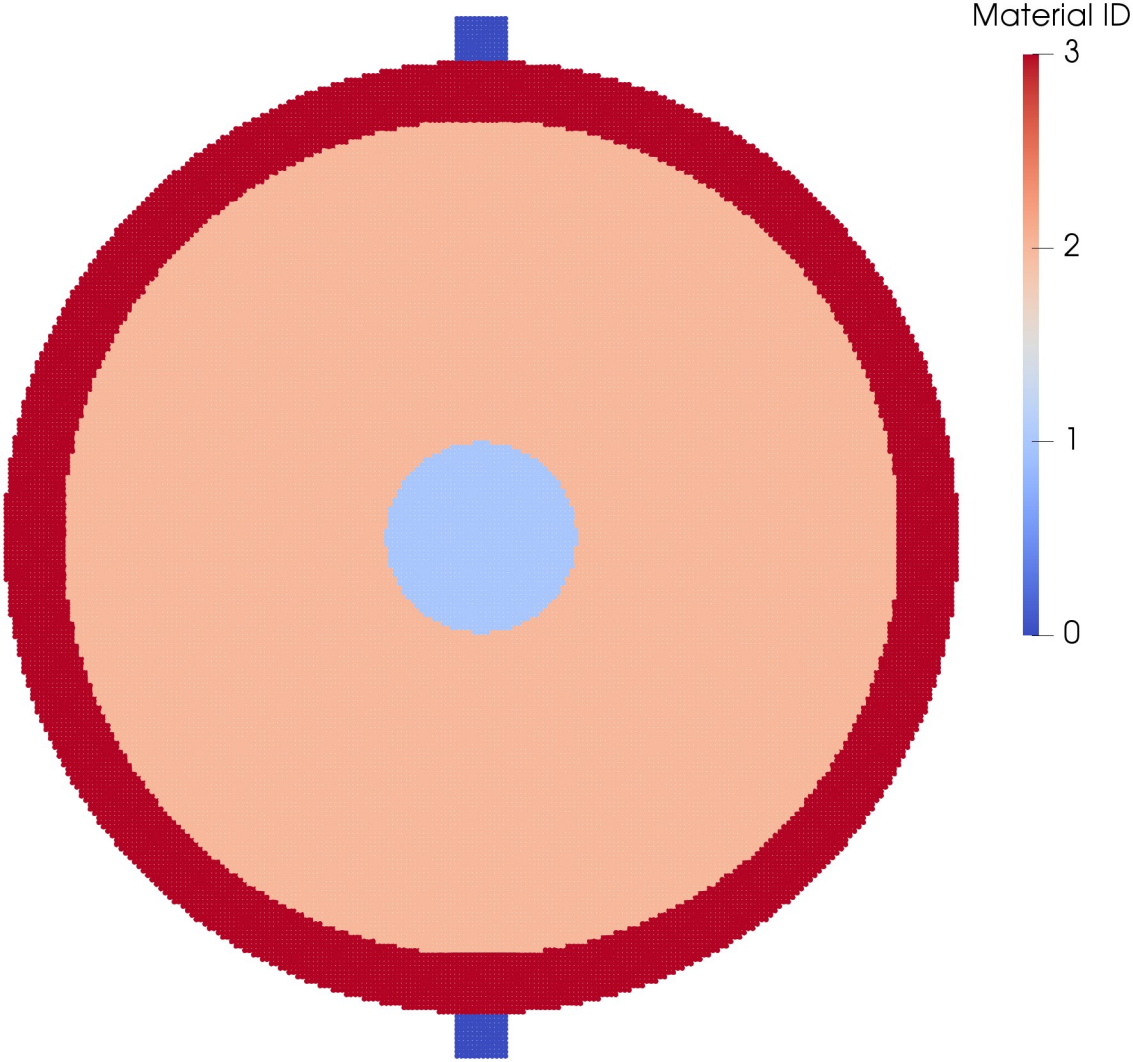
Material distribution for the layered kidney stone Brazil model. Each color represents a section of the stone with slightly different properties due to the specific chemical structure that is informed from the Raman Spectroscopy analysis. The two dark-blue square pistons on the top and bottom edge of the stone are given a velocity condition to drive into the material to induce tensile failure within the center of the stone.

An initial test of MPM and it’s ability to model the damage behavior of stones is to perform the Brazil test on a stone with bulk-averaged properties. This involved compressing a 2D disk of material to failure. The properties of this bulk averaged stone are shown in Table 1. The numerical set-up diagram is shown in Fig 3. This work follows from the analytically verified models shown in [19], the key difference in these simulations is the relative difference between a stone modeled as a single phase, and one modeled as multi-phases. The disk is loaded via two pistons on opposite sides of the disk that compress the material, inducing tensile failure in the center of the material. This loading is similar to that shown in [10] where synthetic kidney stones were tested to failure. The Brazil test is commonly used to measure the tensile strength of brittle materials [19]. In addition to the single phase stones, to highlight the importance of accounting for material distribution in kidney stone modeling, two different stones were modeled. The first took the approach common in the literature of assuming a single phase of material properties throughout the material, the second approach used the insights from the chemo-mechanical measurements, described in 2.1 to create a varied geometrical distribution of the material phases in the material. Both of these stones were loaded to failure using the loading conditions listed in Table 2. The materials properties of the second stone are listed in Table 3. The material distribution of the layered stone is shown in Fig 4.

**Table 1:** Mechanical properties of the bulk-averaged stone for the Brazil test simulation.

Material Property	Value
Density (kg/*m*^3^)	2790
Bulk Modulus (GPa)	25.633
Shear Modulus (GPa)	11.83

**Table 2:** Brazil test simulation parameters.

Simulation Property	Value
No. Particles	36864
Particle Size (m)	0.25 × 10^−3^
Piston Velocity (m/s)	0.1
CFL Number	0.1

**Table 3:** Mechanical properties of the bulk-averaged stone for the Brazil test simulation.

Material Property	Value
Density (outer material) (kg/*m*^3^)	2790
Density (middle material) (kg/*m*^3^)	2790
Density (inner material) (kg/*m*^3^)	2790
Bulk Modulus (outer material) (GPa)	39.96
Bulk Modulus (middle material) (GPa)	22.13
Bulk Modulus (inner material) (GPa)	14.81
Shear Modulus (outer material) (GPa)	18.44
Shear Modulus (middle material) (GPa)	10.22
Shear Modulus (inner material) (GPa)	6.83

**Fig 5:**
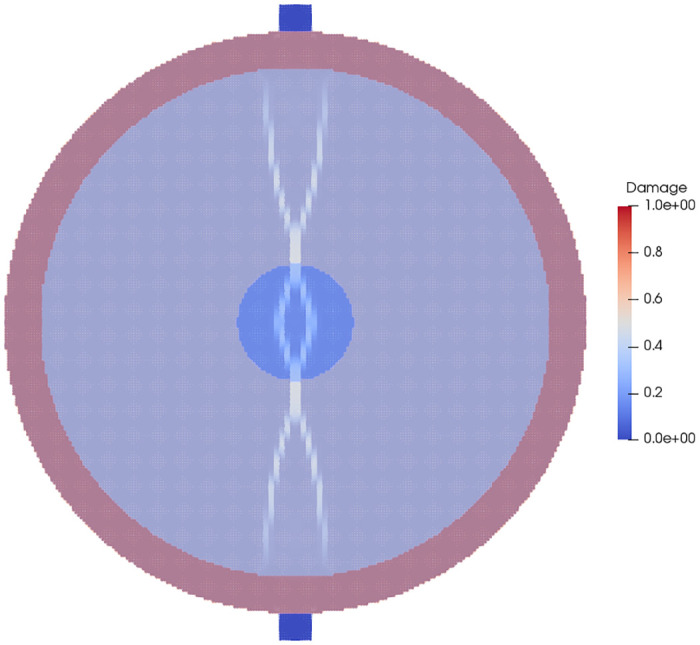
The initial damage of the layered stone with the different material phases overlaid.

**Fig 6:**
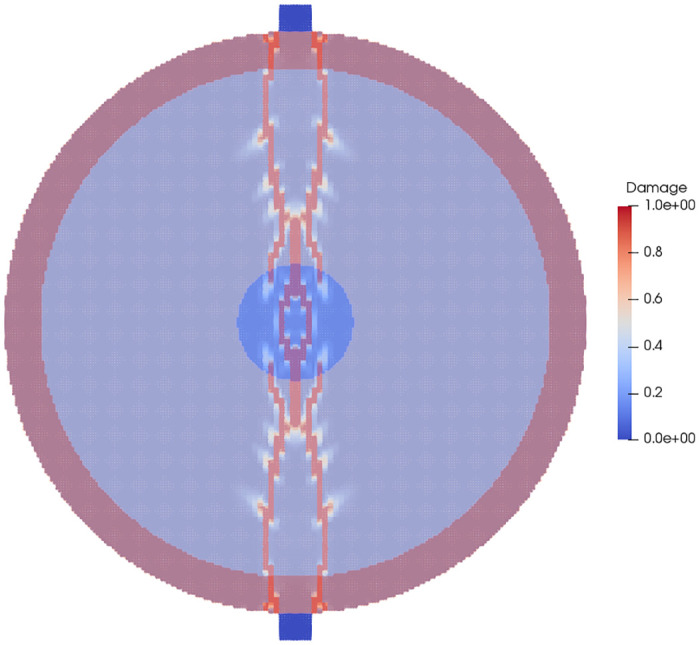
The final damage state of the layered stone with the material phases overlaid.

**Fig 7:**
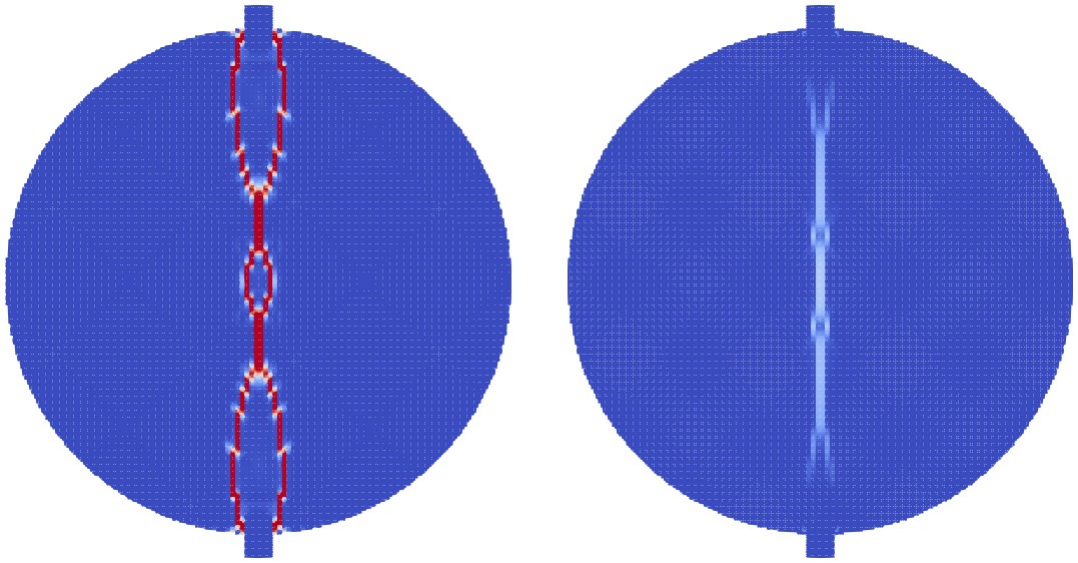
A comparison of the damage profiles of the layered stone (left) and single phase stone (right) at the same point in computation time, the layered stone has accumulated more damage while the single phase stone has only started to fail.

**Fig 8:**
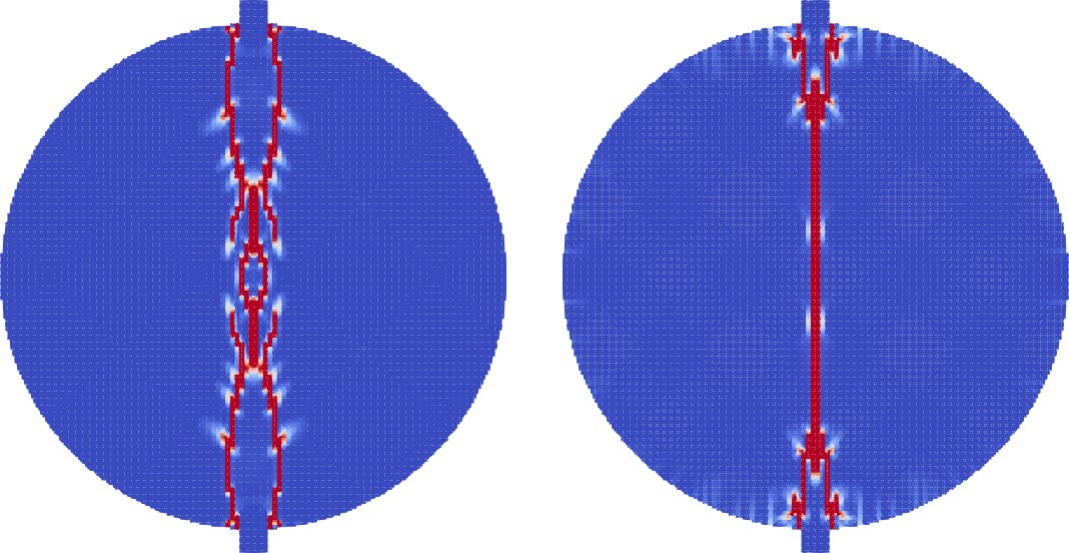
A comparison of the damage profiles of the layered stone (left) and single phase stone (right) at the end of the Brazil test. The layered stone shows a larger amount of accumulated damage for the same loading.

The initial onset of damage and the final damage pattern of the material in the layered disk is shown in Figs 5 and 6, respectively. When the initial damage state of the layered and single phase stones are compared, as in Fig 7 it can be seen that the layered stone, despite having been loaded to the same point as the single phase stone, has already suffered complete damage. In the final damage states of both stones, as shown in Fig 8, the damage patterns of the stones highlight the effect of modeling the stones with their geometrically varied material properties.

This type of loading, however, is not typical of the loads found during kidney stone treatment, therefore these Brazil tests serve merely to show that the different approaches to stone modeling are important when wanting to know where and how a kidney stone might fail. In the next section, the two stones are loaded via shock waves to better approximate the kinds of loading present in real-world treatments of kidney stones.

### 3.2 Damage of kidney stones via shock wave lithotripsy

**Fig 9:**
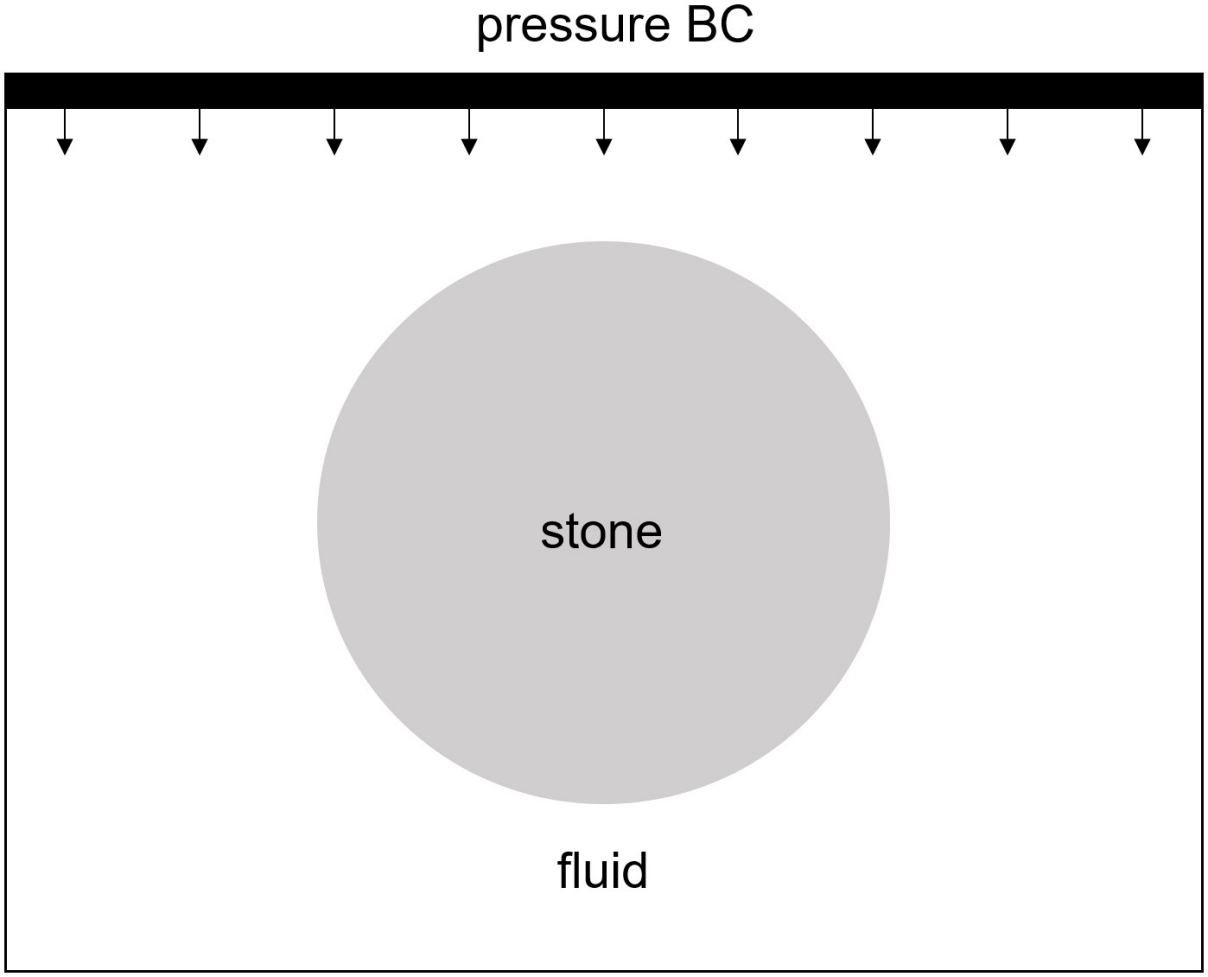
Schematic of the shock wave lithotripsy test showing the location of the pressure pulse, the surrounding fluid and the centered stone.

**Fig 10:**
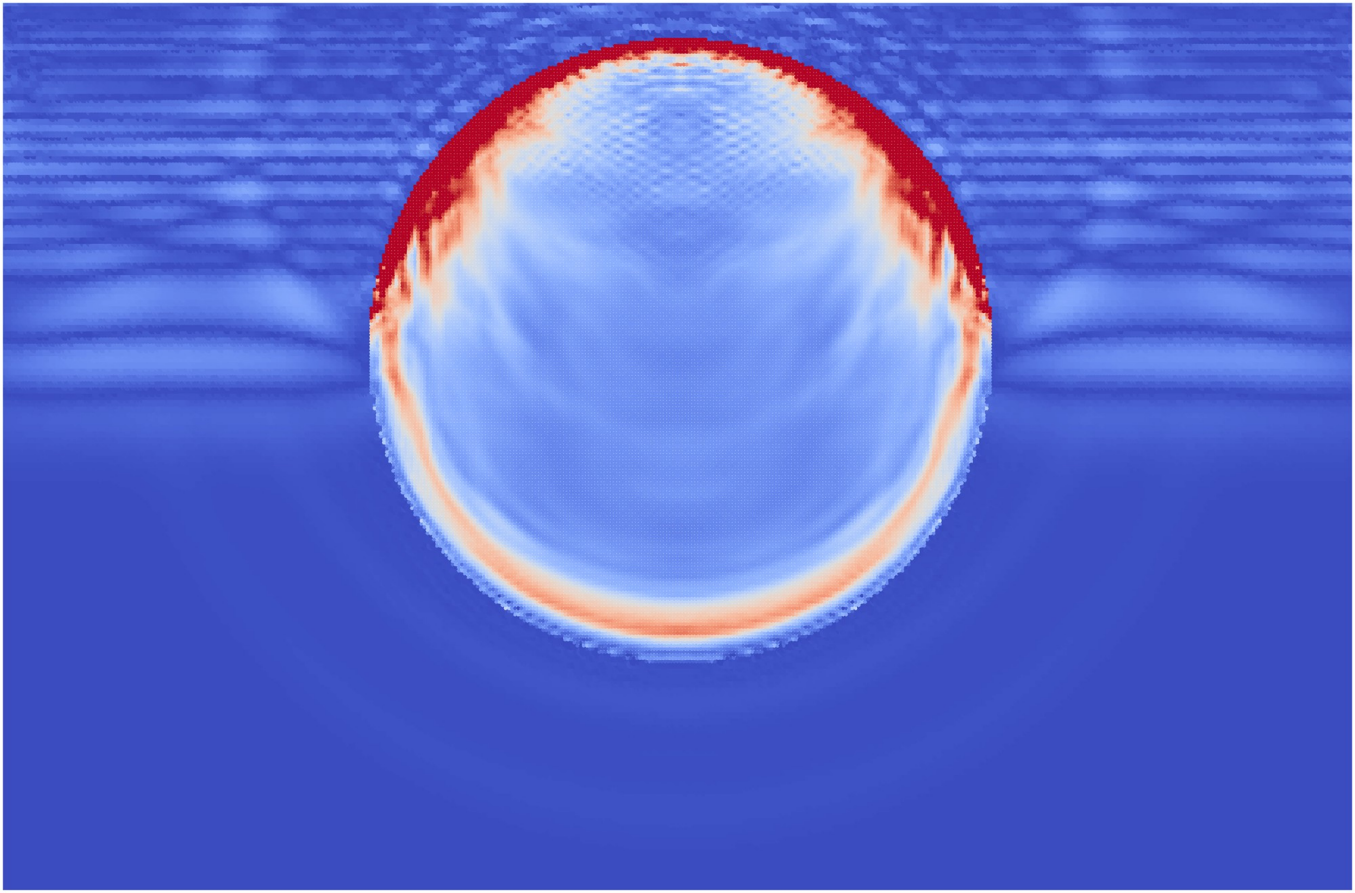
The accumulated damage of the single phase stone when the shock wave (shown as stress in the surrounding fluid) has reached half way along the stone.

**Fig 11:**
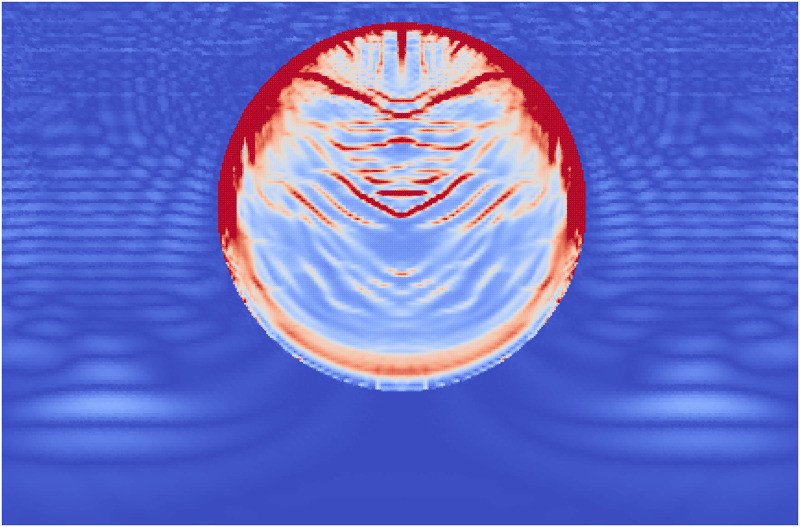
The accumulated damage of the single phase stone when the shock wave (shown as stress in the surrounding fluid) has reached the end of the stone.

**Fig 12:**
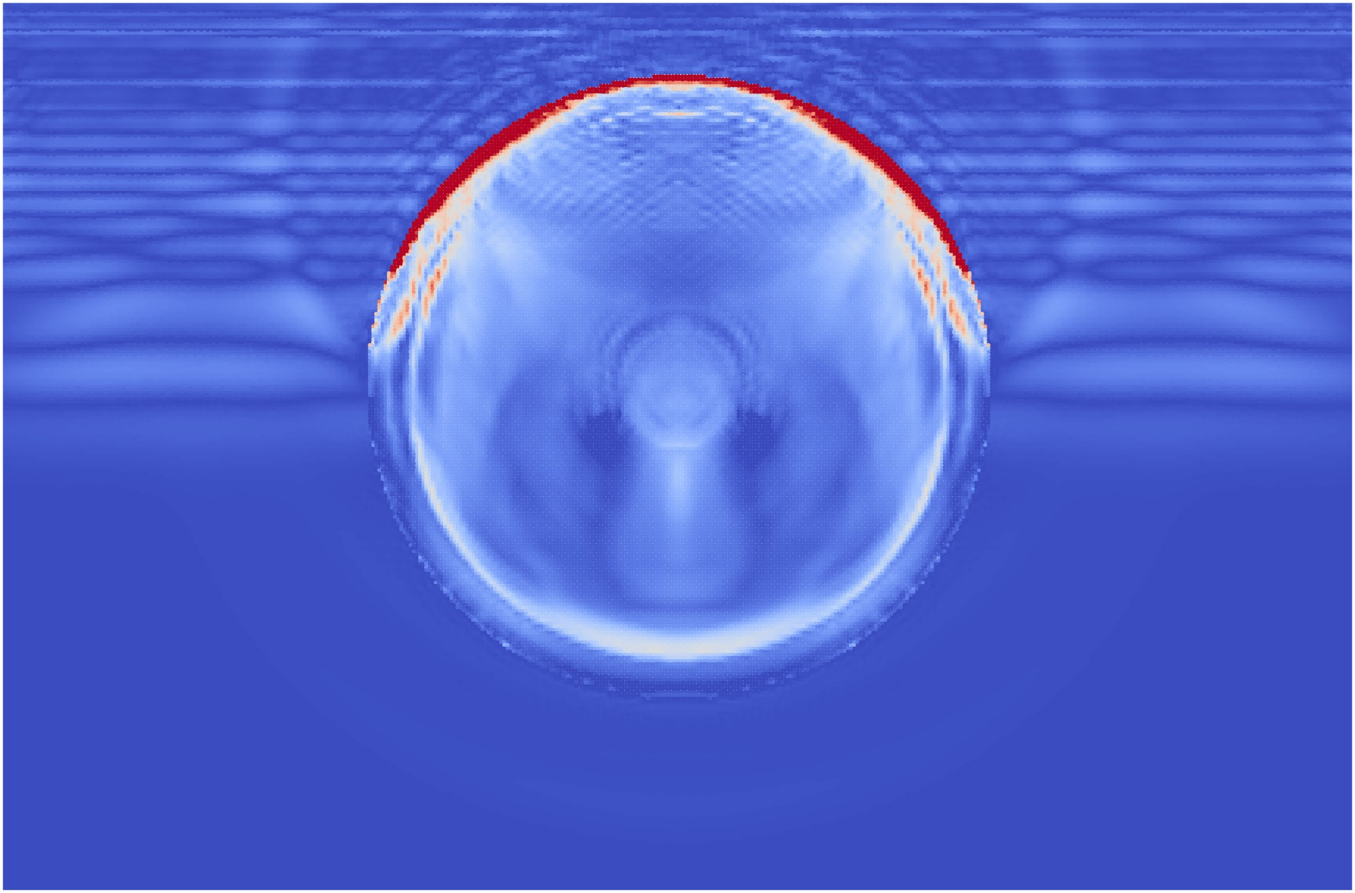
The accumulated damage of the layered stone when the shock wave (shown as stress in the surrounding fluid) has reached half way along the stone.

**Fig 13:**
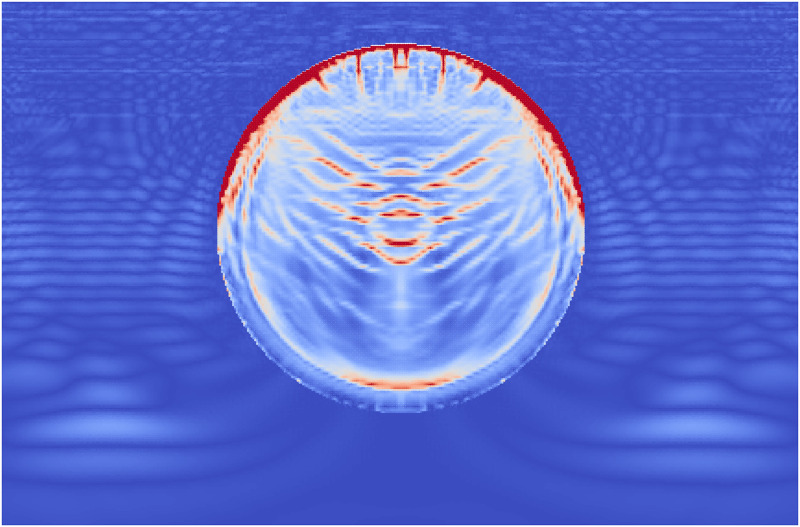
The accumulated damage of the layered stone when the shock wave (shown as stress in the surrounding fluid) has reached the end of the stone.

**Fig 14:**
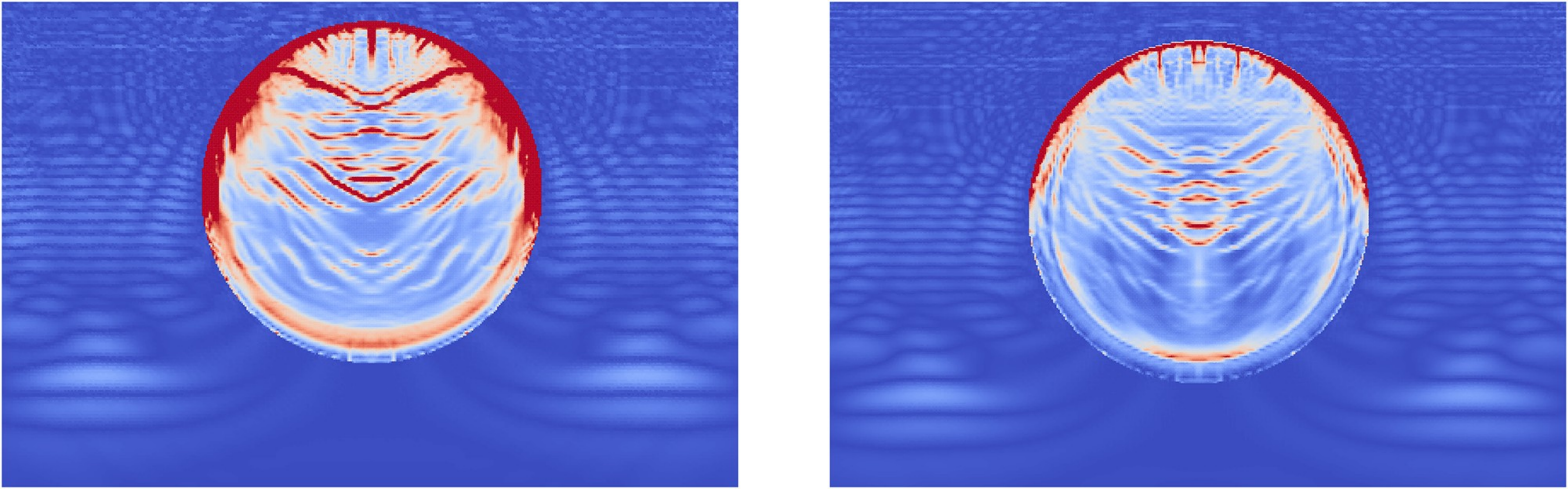
A comparison of the shock wave lithotripsy models for the single phase stone (left) and the layered stone (right) showing the pressure waves in the fluid and the accumulated damage in the stones. The layered stone has accumulated noticeable less damage than the single phase stone.

During Extracorporeal Shock Wave Lithotripsy (ESWL), the patient is positioned on their back with an ultrasonic transducer that is focused on the area of the kidney that is found to be the center of the kidney stone [3]. The stone is subjected to a very high pressure pulse that is transmitted through the skin, the tissue, and the fluids of the kidney stone. This wave passes into the stone and leads to the fragmentation of the stone as the stresses within the stone surpass the material strength. However, the process of how this failure occurs and how to best model it is still an active area of research. The common approach is to model the stress wave as an external wave moving though the computational domain and let it pass into a freely floating stone. This stone is currently always modeled as a single phase material. Given the results from the Brazil test, a shock wave chamber was constructed using MPM and two models were run. The schematic for this setup is shown in Fig 9. The material properties for the two stones were the same as those for the Brazil test (Tables 1 and 3). The fluid was modeled using the properties of liquid water, with the peak pressures and other relevant information in Table 4. To visualize the process of the shock wave impinging on the stones, the fluid material was visualized with the stress waves while the stones visualized the accumulated damage. This way the shock wave progress and the damage to the stone could be visualized in one figure. Fig 10 shows the state of the single phase stone when the shock wave had passed half way along the length of the stone. The front side of the stone shows a layer of damage due to the force of the wavefront impacting on the stone, causing the outer layer of the stone to be completely failed. As the wave reaches the end of the stone, as in Fig 11, the full damage within the stone is reached. A small amount of damage at the rear of the stone from the reflected waves as well as several tensile fractures at the front of the stone can be seen to result from the internal waves of the stone. Also of note are the angled failure lines that result from the interaction of the shear stresses from the edges of the stone as reported in [13, 14, 16] In the Brazil tests, the layered stone failed before the single phase stone, and showed more overall damage. In the ESWL models however, this appeared to be less intuitive. Fig 12 shows the same variables as for the single phase simulations (with the water showing the stress waves and the stone showing the damage). However in Fig 12 the structure of the layered stones, which is the same as in Fig 4 plays an important role. The inner layers serve to reflect parts of the wave and reduce the intensity of the reflections. The result of this is shown in Fig 13 where the total damage of the stone has occurred, and while the locations of failure are comparable, the intensities of the fracturing were lessened. A comparison of the final two stone states is shown in Fig 14 where the importance of including stone geometry is clear. The outer layer of the stone, being stronger than the average bulk properties, protects the stone more and less initial damage on the front side of the stone occurred. Internally, the softer area is targeted with more damage around the center if the stone and with the reflection of waves across material properties, less overall damage is found in the layered stone for the same shock wave.

**Table 4:** Fluid properties and simulation parameters for the shock wave lithotripsy models.

Property	Value
Density (water) (kg/*m*^3^)	1000
Bulk Modulus (water) (GPa)	2.0
Viscosity (water)	1.0 × 10^−3^
No. Particles	175508
Particle Size (m)	0.5 × 10^−3^
Pressure Pulse (MPa)	18
CFL Number	0.025

## 4 Discussion

This work focused on the modeling of kidney stone failure using a novel chemo-mechanical process and a mesh-free computational method to account for the different failure behavior of a stone based on the spatial dependency of it’s material properties. This novel scanning and nano indentation workflow allowed for a detailed phase map to be constructed where the macro-scale properties of the stone could be assigned different spatial locations. This differs from the current approach to modeling stones where a single set of material properties is used to approximate the entirety of the stone. These properties often come from non-kidney stone materials and instead rely on artificially fabricated stones so that the dynamics can be studied more easily. However, accounting for the spatial variation and using properties of real kidney stones is vital in improving kidney stone models. The computational mechanics method, MPM used in this work relies on representing the material as a collection of particles, or material points, which can be used to conform to an arbitrary geometry, allow for multiple phases to be included in a single model, and allow for the initiation and propagation of damage to be calculated with the stone under loading. This is the first use of a particle-based method to analyze the dynamics of fracture growth durisng SWL. This differs from the literature as the majority of computational models are based on grid methods like FEM and FVM. MPM and other meshfree or particle methods have distinct advantages over mesh-based methods in cases of complex geometries, multiple phases, and failure and fragmentation and have been used in other areas of science and engineering for these purposes, this approach allows the fluid and solid to be modeled in a single computational domain, side-stepping the need for any special coupling processes between methods. To observe the effect of material heterogenity in real-world stones, a comparison of a stone modeled in the traditional manner (with the material properties to be considered as bulk properties and applied to a single phase across the entire stone) and a new model where the layers of different phases are explicitly accounted for was performed. The mechanical and failure behavior of these two stones were compared to evaluate the effect of geometry and material distribution. The first test, a Brazil test, is a commonly used method to calculate the tensile strength of a brittle material. In this case, the layered stone was found to fail earlier, for the equivalent loading, to the single phase stone as the center of the stone is loaded more intensely, and with the center of the stone modeled as weaker than the outer layer, this resulted in the layered stone being easier to break than the single phase. While this model is simplified compared to the real-world stone geometries and materials, this result showed the importance of accounting for this difference, even in simple cases. However, in the second case, where a shock wave is sent through a fluid, in this case water, and allowed to impinge on the stones from the outside, the layered stone was found to be harder to break than the single phase stone. This was due to the tougher outer layer initially protecting the inside of the stone, and with the layers of material phases within the stone, the reflection of the stress waves prevented as much focusing as was present in the single phase stone. These results indicate the importance of representing the internal structure of these stone more accurately. The composition of the stone plays a large role in the mechanical response and failure behavior and for a better treatment prediction and understanding, incorporating these components is necessary. While the results and models in this work also take an idealized approach to the distribution of the materials within a kidney stone, even this simple, crude approach is enough to show the inherent differences. In terms of the clinical context, this difference between the compressed stone and the shocked stone highlight the potential for counter-intuitive treatment strategies that may need to be conducted in order to achieve the best results. The workflow shown here can be used to determine which stones are likely to break with the direct shock and those that will need a more sophisticated approach of shock wave treatment (such as shown in [46]) in order to treat. MPM is able to model several waves and continuous growth of fractures [40] and as such can be used to model the progressive damage to a stone in this instance. In the context of the current state of the art analysis of renal calculi simulations, this work offers some advantages, while also still lacking some components that have been used in the study of these stones. In relation to the work in [47], this damage model allows for the growth and partial damaging of sections int he stone, rather that just binary fragmentation, however in contrast to the work of Cao et. al [48], the damage model used in this work was intended for simple brittle failure with values used to approximate stone-like behavior, a more detailed damage model with experimentally derived values for stones would be more appropriate. However the advantage of using MPM in this manner allows the use of more flexible geometric and multi-material setups afforded by the Lagrangian nature of the material points. The work by Zhang et. al [46] showed the effect of multiple pressure wave treatments on the comminution process, this work relied on the single peak pressure wave to ascertain the results. Further work can be done to observe the effects of different wave patterns on the damage process and should be investigated. Finally, the work in this paper focused on the direct interaction between the traveling pressure wave and the effect on the submerged stone. However, as has been shown in several works [49–52] cavitation of bubbles at the surface of the stone can lead to a significant amount of damage in the stone, which was not modeled in this work. While this work shows novel approaches in the field of kidney stone modeling, there are still several limitations present that should be addressed. Firstly the properties used to model the stones were taken from a single stone’s sampling. Ideally a statistical evaluation of the most likely stone constituents should be used to create these synthetic stones to be more applicable in the real-world. In addition, the geometry of the stones were still idealized into concentric circles, which do represent the overall structure shown in many stone types, it is possible that with more refined spatial descriptions of stone properties, different fragmentation behavior will likely be observed. The protein matrix that has also been theorized to aid in the cohesion of the stones was not taken into account and instead a perfect join was assumed within the stone. Additionally, since these stones were idealized as circles with concentric layers, a full 3D model would not have provided sufficient extra information, however with real kidney stones being complex 3-dimensional objects, for the most realistic models, the full 3D profile of the stone with the material properties should be used, as this will also heavily influence the nature of the fracture propagation, which is inherently a 3D process. Finally, to model the damage of these stones there have been several proposed methods, in this work the method was based on previous work of stones with similar properties, however for better results, and to compare with what is closest to occur in the human body, more tests and calibrations of real stones should be conducted so that these results can better inform medical professionals treating these stones.

## 5 Conclusion

A novel workflow combining the computational mechanics method, the Material Point Method (MPM), and a chemo-mechanical spectroscopy technique used to identify different regions of kidney stone material phases is presented. This work explored the application MPM to modeling failure in kidney stones. These stones were modeled as multilayered materials using data from a chemo-mechanical study of lab samples. A better understanding of how these stones are broken apart is a vital piece of knowledge to medical professionals whose aim is to remove these stones safely by breaking them within a patient’s body. While the properties of individual stones are statistically varied, the common elements and proportions are used to generate synthetic stones that are then placed in a digital experiment to observe their failure patterns. To first establish the role of these different material properties, the common Brazil test was used to create a tensile fracture within the center of these stones to observe the effect of stone consistency on failure behavior. The layered and single phase stones were shown to behave very differently depending on the configuration of layer toughness. Next a novel application of MPM was applied in which an ultrasonic wave was produced as a boundary condition, and the pressure was carried by the surrounding fluid, also discretized with MPM, to impact the stones. This numerical modeling of Extracorporeal Shock Wave Lithotripsy (ESWL) revealed how in some cases, the layered outer material protects these stones, emphasizing the importance of accounting for the spatial variation in material strength within these stones.
